# Supplementary material for: Functional network connectivity imprint in febrile seizures
Source: Sci Rep. 2022 Feb 28;12:3267. doi: 10.1038/s41598-022-07173-9 (PMC8885759; doi:10.1038/s41598-022-07173-9)
Supplement: Supplementary file 1 — Supplementary Figures. [file 41598_2022_7173_MOESM1_ESM.docx]

**Functional Network Connectivity Imprint In Febrile Seizures**

Ullas V Acharya ^a#^, Karthik Kulanthaivelu ^a#^, Rajanikant Panda ^a#^,

Jitender Saini ^a^, Arun K Gupta ^a^, Bindu Parayil Sankaran^b^, Kenchaiah Raghavendra ^b^, Ravindranath Chowdary Mundlamuri ^b^, Sanjib Sinha ^b^

ML Keshavamurthy ^c^, Rose Dawn Bharath ^a^ *

^a^ Department of Neuroimaging and Interventional Radiology, National Institute of Mental Health and Neurosciences, Bengaluru-560029, Karnataka, India.

^b^ Department of Neurology, National Institute of Mental Health and Neurosciences, Bengaluru-560029, Karnataka, India

^c^ Department of Paediatric Medicine, Indira Gandhi Institute of Child Health,

Bengaluru-560029, Karnataka, India

# **Equal contributing authors**

***Corresponding author**

Dr Rose Dawn Bharath, D.M.

Professor and Head

Department of Neuroimaging and Interventional Radiology,

Mailing address:

Department of Neuroimaging and Interventional Radiology,

National Institute of Mental Health and Neurosciences, Bengaluru-560029, Karnataka, India

Mobile phone: +919480829651

E-mail: drrosedawnbharath@gmail.com

**Supplementary figures**


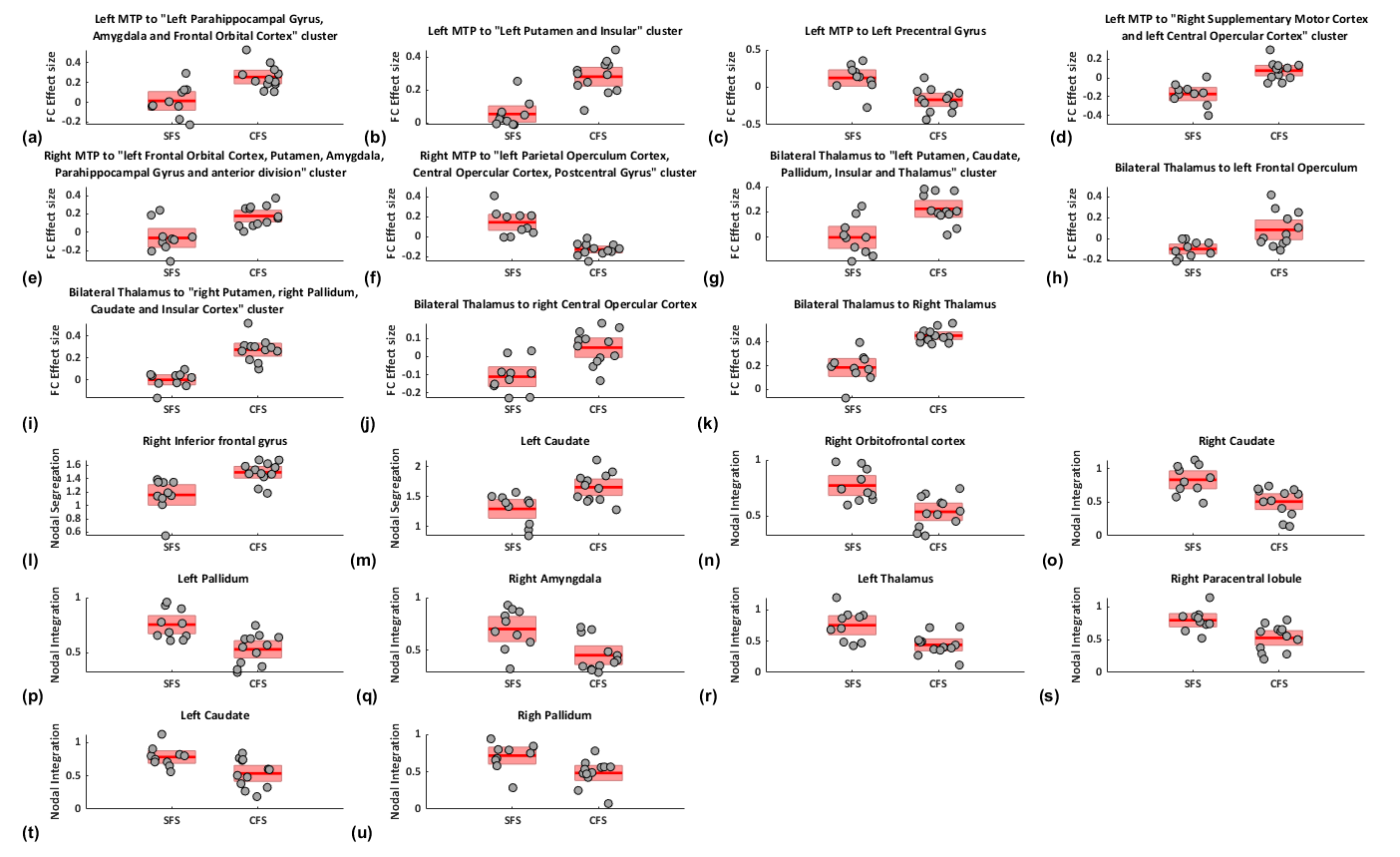


**Supplementary figure 1.** The significant RSFC and graph measures’ of individual subject differences are depicted in a dotted scatter bar plot of SFC and CFS group to highlight low inter-subject variability
